# Relationships Between Emotion Regulation, Social Communication and Repetitive Behaviors in Autism Spectrum Disorder

**DOI:** 10.1007/s10803-021-05340-x

**Published:** 2021-10-28

**Authors:** Agustín E. Martínez-González, Matti Cervin, Jose A. Piqueras

**Affiliations:** 1 Department of Developmental Psychology and Didactics, University of Alicante. Education Faculty, Carretera San Vicente del Raspeig s/n, Campus San Vicente del Raspeig, Edificio Facultad de Educación, Apdo. Correos, PO 99, 03080 Alicante, Spain; 2 Lund University and Skane Child and Adolescent Psychiatry, Lund, Sweden; 3 Department of Health Psychology, Miguel Hernández University of Elche, Edificio Altamira, Avda. de La Universidad, s/n Elche, 03202 Alicante, Spain

**Keywords:** Autism spectrum disorder, Repetitive behavior, Emotion regulation, Social communication, Self-injury, Stereotyped behaviors

## Abstract

The relationship between emotion regulation, social interaction and different types of restricted and repetitive behaviors is poorly understood. In the present study, structural equation modeling based on information about 239 individuals with autism was used to examine whether emotion regulation and social communication were associated with self-injury and stereotyped behaviors. Results showed that poor emotion regulation had a unique association with self-injury while difficulties with social communication was uniquely associated with stereotyped behaviors. Emotion regulation and social communication were strongly associated and self-injury and stereotyped behaviors moderately associated. This implies that these types of behaviors are often expressions of broader negative emotional states in autism. Treatments that help improve coping and social communication strategies may benefit individuals with autism.

## Introduction

Autism spectrum disorder (ASD) is a neurodevelopmental disorder which is characterized by a persistent deficit in social communication and interaction skills, and repetitive and restricted patterns of behavior (APA, 2013). In ASD, restricted and repetitive behaviors (RRBs) are defined as a series of behaviors (e.g., stereotyped, self-injurious, compulsive, ritualistic, sameness, etc.) and activities or interests that manifest regularly and interfere with daily functioning (Bodfish et al., 2000).

Studies have shown that there is a clear relationship between RRBs and emotional states among individuals with ASD as RRBs have been linked to anxiety and stress (e.g., Glod et al., 2019; Russell et al., 2019). Further, RRBs in the form of self-injurious behaviors are closely associated with anxiety in individuals with ASD (Russell et al., 2019). Empirical evidence also supports a relationship between RRBs and emotion regulation difficulties in ASD, which has been considered through the lens of theory of mind (ToM) and executive functioning (e.g., Carter-Leno et al., 2018; García-Villamisar & Rojahn, 2015; Iversen, & Lewis, 2020; Jones et al., 2018). Moreover, difficulties in emotional self-control have been associated with self-injurious behavior and externalizing behaviors in ASD (Carter-Leno et al., 2018; Richards et al., 2017).

*Repetitive Behavior Scale-Revised* (RBS-R; Bodfish et al., 2000) is the most commonly used scale to assess RRBs in adults and children with ASD. The measure has been shown to adequately measure RRBs across the age span and no differences have been found in RRBs according to age (Inada et al., 2015; Lam & Aman, 2007; Martínez-González & Piqueras, 2018, 2019a, 2019b). Further, RBS-R has been shown to be the most accurate predictor of self-injurious behavior in individuals with ASD (Handen et al., 2018).

Social communication difficulties are a defining characteristic of ASD (APA, 2013). Even for individuals that use speech and language to communicate, social communication difficulties are often present as the verbal skill does not meet all of their communication needs (Beukelman & Light, 2020). Thus, as a consequence, social isolation and difficulties in creating and maintaining social networks may appear (Carter et al., 2014; Smith, 2015). Social communication difficulties are a relevant variable for autism research since it has implications in both social and emotional spheres. One of the most frequently used screeners for ASD in the social area is the *Social Communication Questionnaire* (SCQ; Rutter et al., 2003). The SCQ is a commonly used screener for ASD along with other scales (e.g., Autism Diagnostic Observation Schedule (ADOS), Autism Diagnostic Interview—Revised (ADI-R) and SCQ has been shown to be an acceptable screening instrument for autism spectrum disorder (area under the curve = 0.89) (Chesnut et al., 2017) in both children and adults (Berument et al., 1999).

The appearance of repetitive maladaptive behaviors and emotional instability may be factors that could interfere with school functioning and social relationships for individuals with ASD (Inada et al., 2015; Martínez-González & Piqueras, 2018; Rojahn et al., 2013) and studies have indicated that there is a significant relationship between RRBs and deficits in communication (e.g., Jones et al., 2018; Lampi et al., 2020; Tse et al., 2018). Furthermore, it seems that difficulties in social communication and RRBs are closely associated with adaptive behavior problems (Estabillo et al., 2018; Frost et al., 2017; Golya & McIntyre, 2018; Inada et al., 2015; Kojovic et al., 2019; Troyb et al., 2016; Williams et al., 2018). It has also been suggested that poor emotion regulation can be a mode of communication in autism (Nuske et al., 2017; Welsh et al., 2019). However, although there are some studies on the relationship between RRBs and social interaction in ASD, this association is overall poorly understood (Bahrami et al., 2012; Enloe & Rapp, 2014; Joosten et al., 2012; Tse et al., 2018). In fact, there are a limited number of studies that have examined self-injury, stereotyped behaviors, social communication and emotional stability in ASD in tandem (Jones et al., 2018). Such work could help identify individuals who are at the greatest risk for developing self-injurious behavior by identifying strong risk factors (Handen et al., 2018).

One of the concerns of school and mental health professionals is to know the origin and functionality of RRBs. Studies have been carried out from the field of education to biology to study the factors that could be determining factors (e.g., Andreo-Martínez et al., 2018, 2020, 2021; Martínez-González & Piqueras, 2019a; Martínez-González & Andreo-Martínez, 2019). In addition, self-injurious behaviors in ASD pose a risk to physical health and can lead to substantial healthcare costs (Shields et al., 2019). Therefore, it is necessary to analyze the relationships between emotion regulation, social communication, and different types of repetitive behaviors (primarily stereotyped and self-injurious behaviors) among individuals with ASD in order to identify key areas to address in treatment and care (Frost et al., 2017; Fulceri et al., 2016; Inada et al., 2015; Troyb et al., 2016).

The aim of the present study is to examine whether social communication and emotion regulation is associated with stereotyped and self-injurious behaviors in ASD, and whether there is an association between social communication and emotion regulation and stereotyped and self-injurious behaviors, respectively. Importantly, all associations will be estimated within a single model where associations between these factors are analyzed in tandem, which provides an opportunity to outline unique associations.

## Methods

### Participants

An incidental sample of 239 participants was recruited from 18 educational facilities in Spain: 4 special education schools in the Region of Murcia, 2 residential facilities for individuals with ID in the Region of Murcia and Province of Alicante, 7 daycare centers (5 belonged to the Region of Murcia and 2 belonged to the Province of Alicante), 3 early childcare centers (1 belonged to the Region of Murcia and 2 belonged to the Province of Alicante), and 2 regular schools with open classrooms in the Region of Murcia. The participants belonged to differently sized urban areas, and they represented both rural and urban areas. All educational centers had coeducational autism support classrooms except for one private center, which was open only to boys. Public and semiprivate institutions were equally represented in the sample of participating special education centers. All the residential and daycare centers were managed by the associations of individuals with disabilities, which had an agreement with the public administration.

The sample inclusion criteria were delineated based on the DSM-5 diagnostic criteria for ASD (APA, 2013). The subjects had previously been diagnosed by the mental health services and institutions in Spain that are responsible for ascertaining the degree of disability and dependency. We excluded individuals who presented with other diagnoses such as motor disability, multiple disabilities, attention-deficit/hyperactivity disorder, obsessive–compulsive disorder, neurodegenerative diseases, and mental illnesses. We included participants with ASD and ID when ASD was the primary diagnosis. Table 1 shows the sociodemographic and diagnostic characteristics of the sample.

**Table 1:** Sociodemographic characteristics

	Full sample	ASD w ID	ASD w/o ID
N (%)	239 (100%)	154 (64%)	85 (36%)
Age, *M* (*SD*)	13.55 (9.96)	14.92 (11.36)	11.07 (6.01)
Male gender, N (%)	182 (76%)	112 (73%)	70 (82%)

*w* with, *w*/*o* without, *ASD* autism spectrum disorder, *ID* intellectual disability

### Measures

#### Sociodemographic Questionnaire

Lam and Aman’s (2007) sociodemographic questionnaire was adapted for use in the present study. It consists of a series of questions about (a) sociodemographic characteristics (e.g., age, sex, country of birth), (b) the primary diagnosis within the autism spectrum as per DSM criteria (e.g., Asperger’s syndrome, autism, pervasive developmental disorder-not otherwise specified), and (c) comorbidities (e.g., depression, bipolar disorder, anxiety, obsessive–compulsive disorder, ID, schizophrenia; these questions entailed dichotomous response options).

#### Repetitive Behavior Scale-Revised (RBS-R)

The RBS-R is a 43-item scale that measures six different dimensions of repetitive behaviors in individuals with ASD and ID: (a) stereotypic, (b) self-injurious, (c) compulsive, (d) ritualistic, and (e) sameness behaviors (Bodfish et al., 2000). Responses are recorded on a 4-point rating scale that ranges from 0 (repetitive behavior does not occur) to 3 (very severe repetitive behaviors). The assessment of repetitive behavior is based on observations of and interactions with the respondent during the past month. The RBS-R has demonstrated adequate psychometric properties for use with individuals with ASD who belong to different countries (e.g., Fulceri et al., 2016; Georgiades et al., 2010; He et al., 2019; Inada et al., 2015; Martínez-González & Piqueras, 2018, 2019b; Mirenda et al., 2010; Rojahn et al., 2013). The internal consistency of the RBS-R (α = 0.93) is high for most of the subscales: stereotypic (α = 0.86), self-injurious (α = 0.83), compulsive (α = 0.70), ritualistic (α = 0.80), and sameness behaviors (α = 0.88). In this study, we used the two subscales assessing stereotypic (items 1 to 6 of the RBS-R), and self-injurious (items 7 to 14 of the RBS-R) RRBs.

#### Social Communication Questionnaire, SCQ Form B (SCQ-B)

The SCQ-Form B (Rutter et al., 2003; Spanish adaptation by Pereña & Santamaria, 2005) is a scale orientated towards parents or caregivers, with a total of 40 items that determine the possible presence of ASD. It provides an overall total score and three possible additional scores (Social Interaction Problems, Communication Difficulties and restricted, Repetitive and Stereotyped Behaviors). The duration of its administration was 10 min. In the present study, the Form B of the scale was used, which assessed behaviors during the past three months. Scores above 15, the cut-off, suggest that the individual is likely to have ASD and a more extended evaluation should be undertaken. The scale has shown adequate psychometric properties (Pereña & Santamaria, 2005). For this sample, the internal consistency values were: 0.87 for social interaction problems; 0.64 for communication difficulties; 0.78 for restricted, repetitive and stereotyped behavior and 0.90 for the total overall score, but only the social communication difficulties scale was used.

#### Social-Emotional Rating Scale: Leiter-R-Questionnaire

The Social-Emotional Rating Scale-Teacher is included in the Leiter’s International Performance Scale-R (Roid & Miller, 2011). The Social-Emotional Rating Scale evaluates the emotional and regulatory dimension (including Regulatory, Temperament, Reactivity and Adaptation subscales) and the social-cognitive dimension (including Attention, Impulse Control, Activity Level and Social Abilities) in children within an educational context. The test provides scalar scores of each of the sub-scales according to three age groups: from 2 to 4 years, from 4 to 6 years and over 7 years, being used in adolescents and adults (Cederlund et al., 2010). All of them indicate positive aspects of a person’s performance. The psychometric properties of the instrument were also appropriate. The Cronbach’s alphas for this sample were: Attention (0.90); Impulsivity (0.86); Activity Level (0.92); Social Skills (0.90); Regulation (0.79); Temperament (0.79); Reactivity (0.66) and Adaptation (0.84). In this study only the emotional and regulatory dimension was included.

In the analysis we exclusively used the two subscales assessing stereotypic RRBs (items 1 to 6 of the RBS-R), and self-injurious RRBs (items 7 to 14 of the RBS- R); the social communication difficulties subscale from SCQ-B; and subscales for emotional and regulatory dimensions (including Regulatory, Temperament, Reactivity and Adaptation subscales) from the Social-Emotional Rating Scale. The reasons for selecting these scales were the following: (1) the *stereotypic* RRBs (items 1 to 6 of the RBS-R), and *self-injurious* RRBs (items 7 to 14 of the RBS-R) are variables related to externalizing symptoms or indicators of emotional distress (e.g., Carter-Leno et al., 2018; Richards et al., 2017); (2); the *Social communication difficulties* subscale is a scale that focuses on social communication and not so much on problems that exist in social interaction compared to the *Social Interaction Problems* subscale; furthermore, it is a determining variable in adaptation difficulties and repetitive behavior in ASD (e.g., Estabillo et al., 2018; Kojovic et al., 2019); and, finally, (3) the *Social-Emotional Rating Scale—Leiter-R-Questionnaire* is a questionnaire that includes a version for teachers and was considered an ideal instrument to analyze the emotional and regulatory dimension described in the instructions of the same questionnaire (Roid & Miller, 2011).

### Procedures

The present study was approved by the Ethics Committee of the University of Alicante in Spain (reference: UA-2019-10-04). We obtained written informed consent from the parents and caregivers of the participants who were recruited from schools, daycare centers, childcare centers, and residential facilities.

The tests were administered in the educational centers by educational and behavioral science professionals who were knowledgeable about the behaviors of individuals with ASD (e.g., psychopedagogues/educational psychologists, special education teachers, and general psychologists). The first section of the sociodemographic questionnaire, which was an adaptation of the original instrument (Lam & Aman, 2007), was completed by expert psychologists who had relevant information about the diagnoses of the participants who were previously diagnosed by the health center as per the DSM criteria for ASD (APA, 2013). The rest of the research protocol was completed by the professional who had the highest amount of daily contact with the respective participant at the center. All the participating centers underwent a training session that was organized by the researchers who described the purpose of the research study, the tests that were used, and instructions for test administration.

### Data Analyses

Statistical analyses were conducted in R version 1.3.959 using the R library *lavaan*. First, we examined the psychometric properties of each scale separately using confirmatory factor analysis (CFA). This was done to investigate whether we had adequate measurement of the constructs we wanted to examine. We used the following fit indices to evaluate model/data fit: χ^2^, Confirmatory Fit Index (CFI), Root Mean Square Error of Approximation (RMSEA) and Standardized Mean Square Residual (SRMR). RMSEA below 0.06, SRMR below 0.08 and CFI and TLI estimates greater than 0.90 are indicative of acceptable model-data fit; CFI and TLI estimates above 0.95 are indicative of good model-data fit (Hox et al, 2017). A combination of the full set of indices was used to evaluate model fit. We also calculated the internal consistency (alpha) for all factors using the CFA models. Because of the large variation in age, we examined whether there was an association between age and each of the measured scales/factors. This was analyzed by including covariance parameters between age and each factor in the final measurement model. We also analyzed whether there were any gender differences using the same approach. After establishing a sound measurement model, we tested a structural model in which we regressed the stereotypic and self-injurious factors on the social communication and emotion regulation factors.

## Results

### Measurement Model

For RBS-R we first tested the stereotypic and self-injurious factors separately. Each factor showed good CFI/TLI values (> 0.95), but the RMSEA index indicated some problems with fit (values above 0.10). We added correlated residuals for two items for the self-injurious factor and three items for the stereotypic factor. This provided good fit for both scales. When we evaluated fit for both scales in the same model (including the correlated residuals) fit was also good (*X*^2^ [73] = 108.2, *p* for *X*^2^ = 0.005, CFI = 0.99, TLI = 0.98, RMSEA = 0.05, SRMR = 0.08). The internal consistency of both scales was excellent (*as* > 0.90).

For SCQ-B, CFI and TLI indicated adequate fit for the social communication difficulties scale but SRMR and RMSEA were above 0.10. Again, correlated residuals between two items were added and after that addition the model/data fit was excellent (*X*^2^ [8] = 4.4, *p* for *X*^2^ = 0.823, CFI = 1.00, TLI = 1.00, RMSEA = 0.00, SRMR = 0.03) and the scale had adequate internal consistency (*a* = 0.88).

For Leiter-R, a model that included the four emotional and regulation scales had questionable fit. With the addition of correlated residuals between two items, the fit was good (*X*^2^ [163] = 353.9, *p* for *X*^2^ < 0.001, CFI = 0.96, TLI = 0.95, RMSEA = 0.07, SRMR = 0.07) and all of the scales had adequate internal consistency (*a*s: 0.85, 0.86, 0.76, and 0.90).

Finally, we tested a full measurement model that included all of the above-described scales/factors. This model showed good model/data fit (*X*^2^ [714] = 1053.8, *p* for *X*^2^ < 0.001, CFI = 0.96, TLI = 0.96, RMSEA = 0.05, SRMR = 0.09). We also fitted a model where we added a higher-order emotion regulation factor with the Leiter-R scales as indicators. This model showed similar fit (*X*^2^ [725] = 1103.7, *p* for *X*^2^ < 0.001, CFI = 0.96, TLI = 0.96, RMSEA = 0.05, SRMR = 0.10) and we proceeded with the latter model because of the principle of parsimony. No statistically significant associations between included scales/factors and age emerged (all *p*s > 0.09). Females trended to have statistically significantly higher scores on the self-injurious factor than males (standardized covariance coefficient = 0.21, *p* = 0.05). No other gender differences emerged (all *p*s > 0.69).

### Structural Model

**Fig. 1:**
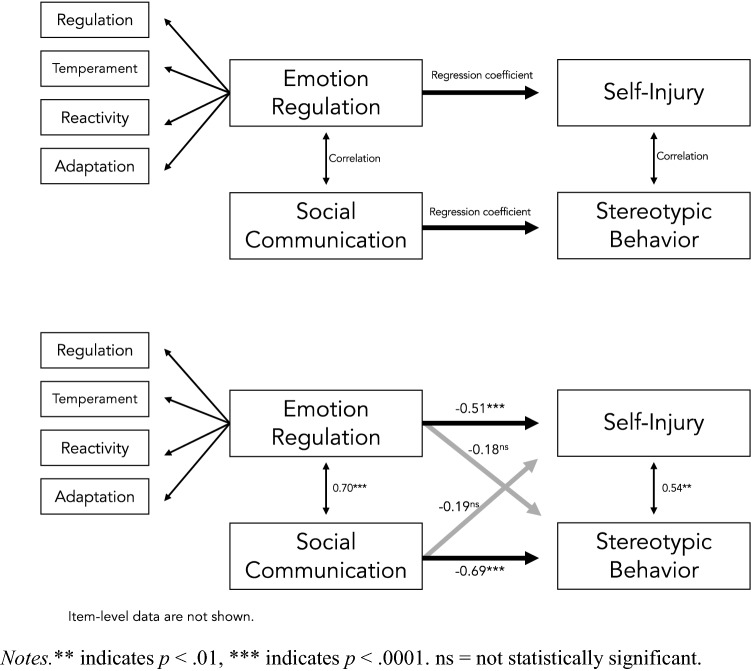
Structural model

Item-level means and standard deviations for the scales that were included in the final measurement model is presented in Table 2. Zero-order Pearson correlations between the scales are presented in Table 3. The top part of Fig. 1 shows the structural model that was fitted. The bottom part of Fig. 1 presents the estimated parameters. In sum, emotion regulation and social communication was markedly associated, and self-injurious and stereotypic behaviors moderately associated. Emotion regulation showed a strong negative association with self-injurious behaviors (i.e., better emotion regulation, less self-injurious behaviors) and social communication a strong negative association with stereotypic behaviors (i.e., better social communication, less stereotypic behaviors). No significant association emerged between emotion regulation and stereotypic behaviors or between social communication and self-injurious behaviors.

**Table 2:** Means and standard deviations across study measures

	*M*	*SD*	*Min*	*Max*
Regulation	1.99	0.60	1	3
Temperament	2.24	0.55	1	3
Reactivity	2.29	0.54	1	3
Adaptation	2.23	0.54	1	3
Communication difficulties	0.40	0.24	0	1
Self-injurious behaviors	0.43	0.43	0	2.75
Stereotypic behaviors	0.56	0.66	0	3

The mean item score is presented; Regulatory, Temperament, Reactivity and Adaptation subscales are included in the *Social-Emotional Rating Scale*—*Leiter-R-Questionnaire*; Communication Difficulties subscale of the *SCQ-B*; Self-injurious and stereotypic behaviors subscales of the *RBS-R*

**Table 3:** Zero-order Pearson correlations between study measures

	2	3	4	5	6	7
1. Regulation	0.62	0.48	0.73	− 0.38	− 0.42	− 0.48
2. Temperament	–	0.44	0.64	− 0.23	− 0.33	− 0.35
3. Reactivity		–	0.60	− 0.44	− 0.35	− 0.42
4. Adaptation			–	− 0.38	− 0.44	− 0.54
5. Communication difficulties				–	0.32	0.37
6.Self-injurious behaviors					–	0.54
7.Stereotypic behaviors						–

Regulatory, Temperament, Reactivity and Adaptation subscales are included in the *Social-Emotional Rating Scale*—*Leiter-R-Questionnaire*; Communication Difficulties subscale of the *SCQ-B*; Self-injurious and stereotypic behaviors subscales of the *RBS-R;* All correlations are statistically significant at the < .01 level

## Discussion

The aim of this study was to analyze the relationship between RRBs (stereotypic and self-injurious behaviors), emotion regulation, and social communication in individuals with ASD. The results indicate that difficulties with emotion regulation have a unique association with self-injury RRBs in individuals with ASD. Thus, when people with ASD have a better capacity to regulate emotion, they tend to engage less in self-injurious behaviors. These results are similar to previous findings showing a correlation between self-injury and externalizing symptoms such as aggressiveness and irritability among individuals with ASD (Carter-Leno et al., 2018; Richards et al., 2017). One possible explanation for this association is the mediating role that executive dysfunction may play in repetitive behavior (García-Villamisar & Rojahn, 2015; Iversen, & Lewis, 2020; Jones et al., 2018).

On the other hand, social communication showed a unique association with stereotypic RRBs. Thus, individuals with ASD with more social communication skills tend to engage less in stereotypic behaviors. These results are in line with previous studies showing a significant association between RRBs and deficits in communication in ASD (e.g., Jones et al., 2018; Lampi et al., 2020; Tse et al., 2018) and point toward a specific link between stereotypic behavior and social communication in ASD. Of note, stereotypic behavior may be more adaptive than self-injury for communicating with the environment. In addition, stereotypic behavior can be a manifestation of anxiety in unpleasant situations (Gabriels et al., 2013).

A strong association was also identified between emotion regulation and social communication. Thus, individuals with ASD with more social communication skills show more emotional stability. These results highlight the importance of emotional self-regulation in order to perform adaptive behaviors (Estabillo et al., 2018; Frost et al., 2017; Golya & McIntyre, 2018; Inada et al., 2015; Kojovic et al., 2019; Troyb et al., 2016; Williams et al., 2018).

Last, a moderate association was found between self-injury and stereotypic behaviors. This indicates that different forms of RRBs are related in ASD and that individuals that engage in one form of RRBs can be expected to also have other forms of RRBs. Previous research has shown that individuals with ASD with more emotional problems and non-adaptive behaviors have more self-injury behaviors and higher levels of anxiety (Russell et al., 2019; Shields et al., 2019). We add to this literature by showing that emotion regulation is uniquely related to self-injury while social communication is uniquely related to stereotypic behavior. However, it is important to note that stereotypic behaviors can be functional and adaptive when they alleviate discomfort (Gabriels et al., 2013). All these results lead us to the “*Continuous emotional state-repetitive behavior in ASD*” theoretical hypothesis which suggests that repetitive behaviors of similarity and stereotypes are associated with lower anxiety levels and increasing adaptation to environment. However, repetitive self-injury behaviors are more associated with higher levels of anxiety and non-adaptive behavior (Martínez-González & Andreo-Martínez, 2020). That is, repetitive behaviors can be conceptualized as a *continuum* that ranges from mild and functional (e.g., stereotypic behaviors) to severe and dysfunctional level which lie at the extreme end of the continuum (e.g., self-injurious behaviors). Thus, self-injury is the behavioral manifestation of a negative emotional state. This behavior would act as an important signal for the detection of emotional distress or even a maladaptive mode of communication. Therefore, this fact has important repercussions for designing treatments that can mitigate self-injury behavior and channel anxiety levels with other types of behaviors, such as stereotypes (Martínez-González & López Gil, 2018).

The present study has several limitations. The cross-sectional data precludes causal claims and even though it is reasonable to assume that communication skills and emotion regulation are factors that cause difficulties with RRBs rather than the other way around, longitudinal designs are needed to better understand causality. Second, it was professionals that worked with and knew the individuals in the study who completed the measures and it is unclear to what extent these professionals report valid observations. Future studies should compare the information from families and professionals. Third, it has been shown that there are differences in the severity of repetitive behavior depending on the comorbidity with ID (Martínez-González & Piqueras, 2019b). In the present study, the ASD groups with and without ID were pooled to be able to conduct the analysis. Future studies should analyze the relationship in the variables studied according to the type of ASD.

The scientific literature has related the severity of RRBs in ASD to factors such as the educational context (e.g., Martínez-González & Piqueras, 2019a; Welsh et al., 2019) and biological bases (Andreo-Martínez et al., 2020). For example, individuals with ASD and gastrointestinal symptoms are at risk for problem behaviors and self-injury (Buie et al., 2010). Thus, the approach to assessment of emotional states should consider gastro-intestinal symptoms (Martínez-González & Andreo-Martínez, 2019, 2020). Different approaches have carried out interventions to improve the emotional state of individuals with ASD. Intervention with probiotics, prebiotics, and fecal microbiota transplantation provides promising results for autism, emotional, and gastrointestinal symptoms (Martínez-González & Andreo-Martinez, 2020). Another traditional intervention such as cognitive‐behavioral therapy (CBT) has reduced negative emotional states in children with high functioning ASD (Perihan et al., 2020; Wang et al., 2021) and in individuals with ASD and intellectual disability (Blakeley‐Smith et al., 2021). The CBT can help improve coping and social communication strategies in individuals with ASD. Further, this type of intervention is necessary during and after the COVID-19 confinement because a high level of repetitive behaviors was found in individuals with ASD (Martínez-González et al., 2021). Nevertheless, it is necessary to have a multidisciplinary perspective in order to understand and influence emotional states in individuals with ASD (Andreo-Martínez et al., 2020; Martínez-González & Andreo-Martínez, 2019). Future studies should consider the sensitivity of measures and heterogeneity of samples. They should also identify RRBs that act as specific predictors of adaptive difficulties. Further, by including biological and neurophysiological variables (e.g., gut microbiota, hormones, short chain fatty acids, gastro-intestinal symptoms, etc.) when studying RRBs in ASD, a further understanding of this phenomena can be achieved.
